# Genome-Wide Identification and Analysis of High-Copy-Number LTR Retrotransposons in Asian Pears

**DOI:** 10.3390/genes10020156

**Published:** 2019-02-18

**Authors:** Shuang Jiang, Xiaoqing Wang, Chunhui Shi, Jun Luo

**Affiliations:** Forestry and Pomology Research Institute, Shanghai Key Lab of Protected Horticultural Technology, Shanghai Academy of Agricultural Sciences, Shanghai 201403, China; jiangshuang@saas.sh.cn (S.J.); wxqshirley666@163.com (X.W.); shichunhui6666@163.com (C.S.)

**Keywords:** copy number, dynamics, insertion time, resequencing, retroelements

## Abstract

A large proportion of the genome of ‘Suli’ pear (*Pyrus pyrifolia*) contains long terminal repeat retrotransposons (LTR-RTs), which suggests that LTR-RTs have played important roles in the evolution of *Pyrus*. Further analysis of retrotransposons, particularly of high-copy-number LTR-RTs in different species, will provide new insights into the evolutionary history of *Pyrus*. A total of 4912 putative LTR-RTs classified into 198 subfamilies were identified in the ‘Suli’ pear genome. Six Asian pear accessions, including cultivars and wild species, were resequenced. The comparison of copy number for each LTR-RT subfamily was evaluated in *Pyrus* accessions, and data showed up to four-fold differences for some subfamilies. This contrast suggests different fates for retrotransposon families in the evolution of *Pyrus*. Fourteen high-copy-number subfamilies were identified in Asian pears, and more than 50% of the LTR-RTs in the genomes of all *Pyrus* accessions were from these 14 identified LTR-RT subfamilies. Their average insertion time was 3.42 million years ago, which suggests that these subfamilies were recently inserted into the genome. Many homologous and specific retrotransposon insertion sites were identified in oriental and occidental pears, suggesting that the duplication of retrotransposons has occurred throughout almost the entire origin and evolution of *Pyrus* species. The LTR-RTs show high heterogeneity, and their copy numbers vary in different *Pyrus* species. Thus, our findings suggest that LTR-RTs are an important source of genetic variation among *Pyrus* species.

## 1. Introduction

Transposable elements (TEs) are mobile sequences that use different enzymatic strategies, such as reverse transcriptase, transposase and helicase, to move and insert in all eukaryote genomes [1]. These elements could create insertion mutations and constitute a high percentage of plant genomes [2]. Two main classes have been identified: Class I retrotransposons and Class II transposons [2]. Retrotransposons flanked by long terminal repeats (LTR-RTs) are Class I elements that undergo replicative transposition, and they have been widely investigated in plants due to their distribution and contributions on genome organization [3,4]. Recently, LTR-RTs were reported to change the size of plant genomes. In *Oryza australiensis*, the transposition of retrotransposons has led to a rapid two-fold increase in genome size over the last 3 million years, suggesting that the rapid amplification of LTR-RTs plays a major evolutionary role in genome expansion [5]. In contrast, a larger number of partial retrotransposons have been identified in rice genomes, suggesting that some pathways (e.g., illegitimate recombination) must exist for removing retrotransposons, corresponding to a rapid reduction in genome size [6].

LTR-RT elements exhibit two flanking LTR sequences [1]. The internal sequence is typically composed of two protein domains of *GAG* and *POL* for replication. The *GAG* domain is present in structural proteins related to the maturation and packaging of retrotransposon RNA, and the *POL* domain includes genes coding for the protease, reverse transcriptase and integrase enzymes. There are other conserved sequence motifs including the primer-binding site, target site duplications and a polypurine tract, which are also essential for the replication of retrotransposons [7]. The primer binding site is a complementary hybridization partner for a transfer RNA acting as a primer for the reverse transcriptase [8,9]. Based on the order of reverse transcriptase and integrase in *POL* genes, LTR-RTs can be classified into two superfamilies: Ty1-*copia* and Ty3-*gypsy*. Retrotransposons have many different families in plants, and they display a high level of sequence diversity [10]. The insertion of retrotransposons within or near transcriptionally active regions can cause mutations by disrupting genes, altering gene expression levels, or driving genomic rearrangements [11,12]. In grape, a retrotransposon inserted into a *myb*-related gene caused pigmentation loss [13]. In blood orange, a retrotransposon inserted into upstream of an anthocyanin biosynthesis-related gene caused cold-dependent color formation in fruit [14]. The insertion of the *Taigu* retroelement into the gene *Ms2* promoter results in *Ms2* expression that confers male sterility in wheat [15].

*Pyrus* L. (pear) is classified in the subtribe *Malinae* of the tribe *Maleae* [16], and may have originated in the mountainous regions of western and southwestern China and gradually spread to European regions [17]. Based on geography, *Pyrus* is divided into two groups: occidental and oriental pears [18]. The oriental China region contain 13 native species of *Pyrus*, including the cultivated species *P. pyrifolia* and the wild pears *P. calleryana*, *P. pashia*, and *P. betulaefolia* [19]. Their evolutionary history is still unclear [20]. *P. communis* is the major cultivated species of occidental pear, and it has been widely produced throughout Europe, North and South America, and Africa. Recently, the whole genomes of *P. pyrifolia* Chinese white pear ‘Suli’ and *P. communis* ‘Bartlett’ were sequenced [21,22]. The assembled ‘Suli’ genome consists of 2103 scaffolds with an N50 of 540.8 kb, totaling 512.0 Mb with 194× coverage. The ‘Bartlett’ genome, which consists of 142,083 scaffolds with an N50 of 6569 bp, totaling 577.0 Mb with 11.4× coverage, was poorly assembled. The scaffolds revealed that a huge part of the *Pyrus* genome is retrotransposon-derived, and 42.4% of the ‘Suli’ genome consisted of LTR-RTs [22]. A variable percentage of retrotransposons can be observed when we compare the genomes of different plant groups, such as *Oryza* (25.8%), *Glycine* (36.2%), *Malus* (37.6%) and *Prunus* (18.6%) [23,24]. It suggested that the duplication of retrotransposons was different among species. In the genomes of cultivated *Pyrus* species, the retrotransposons insert into many loci, but the homologous insertions were rarely found in the genomes of wild *Pyrus* species [25,26]. This finding suggested that retrotransposons might be involved in the evolution of *Pyrus* genomes. Therefore, research on the dynamics of retrotransposons in *Pyrus* species, particularly the copy number variety of LTR-RTs, will enhance the current understanding of the evolutionary history of *Pyrus*.

Previous research showed that a high percentage of the ‘Suli’ genome was LTR-RTs, but the characteristics of these LTR-RTs and their distribution in other *Pyrus* species remain unclear. In this study, we predicted LTR-RTs in the ‘Suli’ genome data and investigated their copy numbers in six *Pyrus* accessions, including cultivars and wild species. Whole-genome resequencing data was used to evaluate the dynamics of LTR-RTs in the evolution of Asian pears. LTR-RT subfamilies with high copy numbers were isolated, and their insertion times and sites were further investigated; these results might provide new insights into the genome structure and the evolution of *Pyrus*.

## 2. Methods and Materials

### 2.1. Plant Materials and DNA Extraction

The plant materials sequenced in this study consisted of six *Pyrus* accessions (three cultivars from *P. pyrifolia*, ‘Suli’, ‘Qiushui’ and ‘Zaoshengxinshui’, and three wild species, *P. pashia*, *P. calleryana* and *P. betulaefolia*). Genomic DNA was extracted from the young leaves of each specimen using the CTAB protocol according to Doyle and Doyle [27].

### 2.2. The Genome Data

The whole-genome assembly data of *P. pyrifolia* Chinese white pear ‘Suli’ and *P. communis* ‘Bartlett’ 1.0 were downloaded from NCBI (AJSU00000000) and Genome Database for *Rosaceae*, respectively. The chromosome data was downloaded from the Center of Pear Engineering Technology Research (http://peargenome.njau.edu.cn/).

### 2.3. Identification and Annotation of LTR-RTs

LTR-harvest [28] was used to predict full-length LTR-RTs from the whole-genome data of ‘Suli’ according to Jiang et al. [26]. The similarity of the two LTR sequences was set to 85% to obtain a large number of retrotransposons. An in-house Perl script was used to translate all isolated LTR-RTs to protein sequences in all six potential reading frames. Four gene models (*GAG*, *PF03732*; integrase, *PF00665*; reverse transcriptase, *PF00078* (*gypsy*) and *PF07727* (*copia*)) were downloaded from the Pfam. Each gene model was used to search all proteins translated from retrotransposons with HMMER3.0 software [29]. The pseudo-LTR-RTs with “No gene models found” were deleted. The remaining retrotransposons were submitted to CENSOR in Repbase [30]. The incomplete or merged retrotransposons were deleted, and the remaining LTR-RTs were classified into different subfamilies based on the search results in Repbase.

### 2.4. Whole-Genome Resequencing of Pyrus Species

High-quality genomic DNA was randomly interrupted by ultrasound. DNA fragments ranging from 150~800 bp were recovered by electrophoresis. T4 DNA Polymerase, Klenow DNA Polymerase and T4 PNK were used to repair cohesive ends to blunt ends. The 3′ end of DNA fragment was amended including an “A” base and ligated to adaptors, including a 5′ end T base. All fragments were recovered by electrophoresis and then paired-end sequenced by an Illumina HiSeq 2500 (San Diego, CA, USA). The adaptors and low-quality reads with more than 20% bases of quality value ≤10 in raw sequence data in FASTQ format were filtered by Trimmomatic. Only clean reads were used in the subsequent analysis. The clean read data were deposited in the Genome Sequence Archive in the BIG Data Center (http://bigd.big.ac.cn/gsa), and the accession numbers are CRR019693-CRR019697. Three randomly selected sequencing datasets of ‘Suli’ (SRR609906, SRR609907 and SRR609912) were downloaded from the SRA database in NCBI.

Sample Availability: The SRA data of whole genome resequencing was deposited in Genome Sequence Archive in BIG Data Center (http://bigd.big.ac.cn/gsa), and the accession numbers are CRR019693-CRR019697. The code of all Perl Scripts was deposited in Baidu cloud (https://pan.baidu.com/s/1f0HVCAif9KQE5gerqBK_vw, password: 38b1.).

### 2.5. Evaluation of the Relative Copy Numbers of LTR-RTs

Bowtie1 was used with default parameters to map all reads from the resequencing data to all identified LTR-RTs and the ‘Suli’ genome [31]. The numbers of reads mapped to the 4,912 LTR-RTs and the ‘Suli’ genome were calculated. For each retrotransposon, reads per kilobase per hundred thousand mapped pear reference genome (‘Suli’ genome) reads (RPKM) were evaluated. An in-house Perl script was used to count mapped reads in the results of Bowtie1. Paired-end sequencing involves sequencing both ends of a fragment. The LTR-RTs were inserted into the genomes, so the resequencing reads might proceed from the LTR sequences and their flanking regions. Paired-end mapping would lose some mapped data in short LTR-RTs and was thus not suitable in this study. The paired-end data were first separated into two single-end datasets and then analyzed by Bowtie1. The average RPKM value of the two single-end data represented the final value for each LTR-RT. The box plot was drawn by using the R programming language. The sum of the RPKM values of the LTR-RTs from one family represented the RPKM of that family. The absolute value of |log2Ratio| ≥ 2 was set as the threshold to determine the significance of the difference of RPKM value among pear accessions.

### 2.6. The Insertion Times and Classifications of High-Copy-Number LTR-RT Subfamilies

ClustalW was used to align the two LTRs of each isolated retrotransposon [32], and genetic divergence between the two LTRs was estimated using Mega 7 software [33]. The insertion time (*T*) was estimated for each LTR-RT using the formula

*T* = *k*/2*r* (1)

where *k* is the divergence between two LTRs and *r* is the substitution rate of 4.72 × 10^−9^ substitutions/site/year [34]. The amino acid sequences of reverse transcriptase in the *copia* and *gypsy* retrotransposons were separately aligned to those of known transposable element (TE) families, including *Ivana*, *Ale*, *Maximus*, *TAR*, *Bianca*, and *Angela* for *copia* elements and *Tekay*, *CRM*, *Athila*, *Tat*, *Galadriel*, and *Reina* for *gypsy* elements using ClustalW, and a neighbor-joining tree was generated using Mega 7 software based on their genetic distances.

### 2.7. Genomic Distribution of High-Copy-Number LTR-RT Subfamilies

The chromosome data of ‘Suli’ and ‘Bartlett’ were used to display the distribution of high-copy-number LTR-RTs. We used an in-house Perl script to analyze the distribution of LTR-RTs on the chromosomes. The assembled 378 Mb of 17 ‘Suli’ chromosomes was separated into 378 nonoverlapping 1-Mb windows, and the observed copy number of LTR-RTs in each window was calculated by discontiguous megablast searching. The same method was used for the ‘Bartlett’ chromosomes. Circular genome data visualization was performed in Circos [35].

### 2.8. The Homologous and Specific Insertion Sites in the ‘Suli’ and ‘Bartlett’ Genomes

The LTR sequences of the isolated LTR-RTs were mapped to the ‘Suli’ and ‘Bartlett’ genomes using BLASTN. The position of each LTR-RT was found. We isolated 200 bp upstream and downstream of LTR-RT in both of ‘Suli’ and ‘Bartlett’. An in-house BioPerl script was used to extract the nucleotide sequences of the LTR flanking regions. The comparison of LTR flanking regions between ‘Suli’ and ‘Bartlett’ was based on BLASTN, and the homologous and specific insertion sites between the two *Pyrus* genomes were calculated.

## 3. Results

### 3.1. Prediction and Classification of Retrotransposons in the Pyrus Genome

To identify a large number of LTR-RTs, 85% similarities among 5′ and 3′ LTR sequences were used to predict retrotransposons by LTR-harvest, which might avoid bias toward recently inserted retrotransposons. Two *Pyrus* genomes were used in the prediction. A total of 11,259 putative full-length LTR-RTs were identified in the ‘Suli’ genome (N50 value, 540.8 kb). No more than 100 full-length LTR-RTs were identified in the ‘Bartlett’ genome owing to an insufficient sequencing depth and low N50 value (6569 bp); thus, LTR-RT prediction in this genome was not pursued further. To delete some pseudo-retrotransposons, we searched all identified LTR-RTs from the ‘Suli’ genome for the conserved protein domains GAG, reverse transcriptase and integrase. A total of 4912 putative LTR-RTs (43.6%) contained at least one of these domains, and they were searched as query sequences in the Repbase database. The result showed that some of them were mapped to the partial sequence of one reference retrotransposon, and some were mapped to more than two reference retrotransposons (Supplementary Figure S1). A total of 1276 LTR-RTs were mapped to only one full-length retrotransposon in Repbase, and their sequence similarity was more than 70%. Annotation showed that these LTR-RTs were classified into 198 subfamilies (Supplementary Table S1) and that most of them were isolated from the genomes of *Pyrus* (*PX*), *Malus* (*MAD*) and *Prunus* (*PRU* and *PPE*).

### 3.2. Whole-Genome Resequencing in Pyrus Species

The whole genomes of five Asian *Pyrus* accessions (two cultivars of *P. pyrifolia*: ‘Zaoshengxinshui’ and ‘Qiushui’; three wild species: *P. pashia*, *P. calleryana*, and *P. betulaefolia*) were resequenced at a 12× depth by Illumina HiSeq. A total of 306.0 M clean reads (Q20 > 96%) and 50 M for each sample were obtained (Table 1). In addition, three genome-sequenced data sets of *P. pyrifolia* Chinese white pear ‘Suli’ (296.7 M) were downloaded from the SRA database in NCBI and included in the analysis. Bowtie was used to map all reads to the reference genome of ‘Suli’ and 4912 isolated putative LTR-RTs separately (Table 1). In mapping the reference genome reads, 71.4% reads from the sequenced data in ‘Suli’ were mapped to its own genome. In the other two cultivars of *P. pyrifolia* (‘Qiushui’ and ‘Zaoshengxinshui’), 50.9% and 48.9% of reads were mapped to the reference genome, respectively. The percent of mapped reads in the three wild accessions (*P. pashia*, *P. calleryana* and *P. betulaefolia*) ranged from 41.9% to 45.3%. The ratio of the number of reads mapped to the 4912 LTR-RTs to the number of reads mapped to the reference genome revealed the percentage of LTR-RTs in the genomes of the *Pyrus* accessions. Approximately 23.8% of the reads in ‘Suli’ were mapped to the isolated LTR-RTs (Table 1). In the five sequenced oriental *Pyrus* accessions, the percent of mapped reads ranged from 22.4% to 35.1%, and the largest percent, 35.1%, was found in *P. betulaefolia* (Table 1).

### 3.3. Variable Relative Copy Numbers of LTR-RT Subfamilies in Pyrus Species

**Figure 1:**
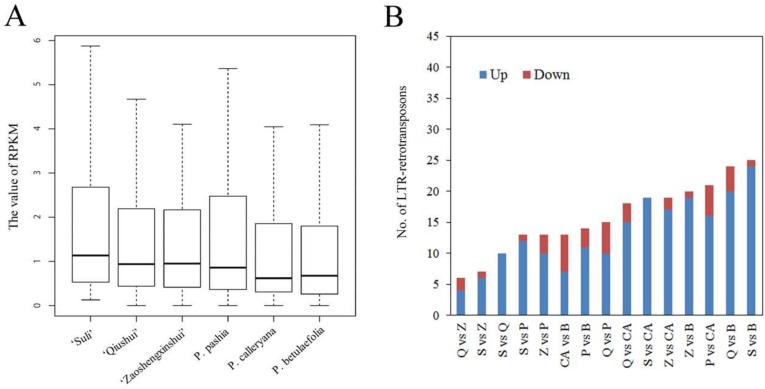
The RPKM values of all 198 LTR-RT subfamilies in *Pyrus* species. (**A**) Boxplots of all values without outliers. The bottom and top boundaries of each box are the first and third quartiles, and the bold lines within individual boxes are the medians, referred to as the second quartiles. (**B**) Pairwise comparison of RPKM values in six *Pyrus* accessions (|log_2_Ratio| ≥ 2). S, ‘Suli’; Q, ‘Qiushui’; Z, ‘Zaoshengxinshui’; P, *P. pashia*; CA, *P. calleryana*; and B, *P. betulaefolia*.

The copy numbers of 198 LTR-RT subfamilies were evaluated in different *Pyrus* accessions. In this study, the RPKM value was selected to represent the relative copy number of LTR-RTs. The number of reads mapped to each LTR-RT was calculated, and then divided by the number of mapped reference genome reads. To be an RPKM value, the result should then be multiplied by a hundred million. This value also corresponded to the sequencing depth, which represented the real relative copy number of each LTR-RT. According to the heterogeneity of LTR-RTs in one family, we first calculated the RPKM values of 1276 LTR-RTs (Supplementary Table S1), and the sum of the RPKM values of the LTR-RTs from one family represented the relative copy number of that family. The RPKM values ranged from 0 to 276.6 (Supplementary Table S2). Some LTR-RT subfamilies in *Pyrus* accessions showed that no reads were mapped to the identified LTR-RTs, and the RPKM value was 0, which suggested that these subfamilies were not present or were rarely found in the genome of this species. For all LTR-RTs, *P. betulaefolia* had the highest RPKM value among the six *Pyrus* accessions (Table 2). ‘Qiushui’ and ‘Zaoshengxinshui’ had low RPKM values. For each LTR-RT subfamily, the RPKM value differed markedly both within and between *Pyrus* species (Figure 1A, Supplementary Table S2). The median RPKM value of the LTR-RTs in all accessions was ranged from 0.62 to 1.13 (Figure 1A). In each LTR-RT subfamily, a large difference in RPKM value between accessions was recorded. The difference in the RPKM values of 25 LTR-RT subfamilies (12.6%) between ‘Suli’ and *P. betulaefolia* was more than 4 times (Figure 1B). Only 6 retrotransposons (3.2%) showed 4-fold differences in RPKM value between ‘Qiushui’ and ‘Zaoshengxinshui’.

The actual copy numbers of LTR-RTs could be identified from the assembly data of the ‘Suli’ genome, such as the five copies found in *Gypsy-7_PX* and one copy in *Gypsy-46_Mad* (Supplementary Table S1). In this study, the RPKM value of *Gypsy-46_Mad* was 24.83 in ‘Suli’, which was higher than the value of *Gypsy-7_PX* (RPKM, 1.04 in ‘Suli’) (Supplementary Table S2). The number directly calculated from the genome assembly data could be affected by LTR-RTs that had lost some part of their sequences and by the genome assembly method. These LTR-RTs were ignored in the prediction; thus, in the current genome assembly method, the same reads were overlapped and ignored in the assembly process, suggesting that some high-copy-number LTR-RTs with highly similar members were assembled into one or a few sequences. The RPKM value was better than the direct calculation of copies from genome assembly data to represent the actual copy number of LTR-RTs. To test the reproducibility of RPKM value between samples, three genome sequencing datasets from ‘Suli’ (SRR609906, SRR609907 and SRR609912) were analyzed (Supplementary Table S3). The results showed that the correlation in pairwise comparison was high (R^2^ > 0.99, Supplementary Table S3).

### 3.4. High-Copy-Number LTR-RT Subfamilies in Pyrus Species

**Figure 2:**
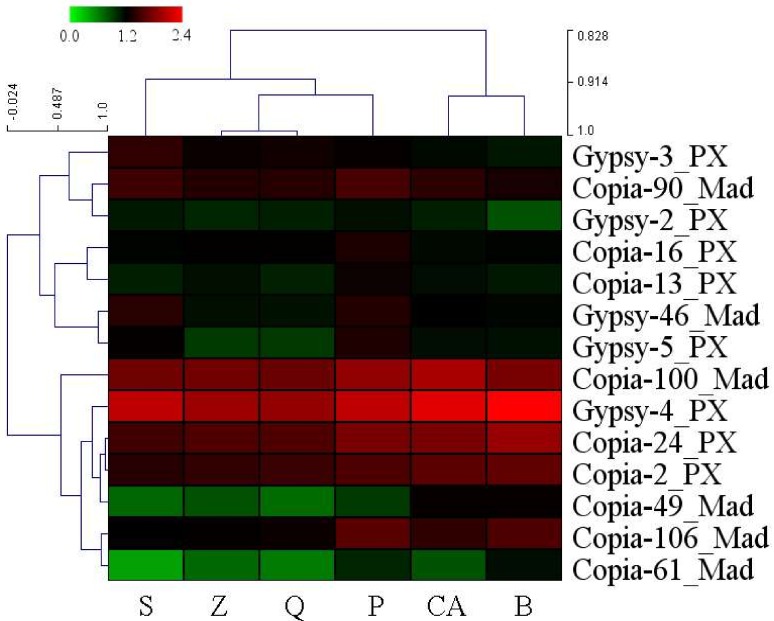
The heatmap of RPKM values in the 14 high-copy-number LTR-RT subfamilies. Data were log_10_-transformed. S, ‘Suli’; Q, ‘Qiushui’; Z, ‘Zaoshengxinshui’; P, *P. pashia*; CA, *P. calleryana*; and B, *P. betulaefolia*. The hierarchical clustering of LTR-RT subfamilies and samples were showed in the **left** and the **top.**

All 198 LTR-RT subfamilies were ordered based on RPKM value, from largest to smallest in all six *Pyrus* accessions. The top 10 LTR-RT subfamilies in each accession were defined as high-copy-number subfamilies. A total of 14 LTR-RT subfamilies were obtained after decreasing duplication from six *Pyrus* accessions (Supplementary Table S4). Nine subfamilies were *copia*-type retrotransposons, and five subfamilies were *gypsy*-type retrotransposons. The RPKM of the 14 identified LTR-RT subfamilies accounted for more than half of the total RPKM of the 198 LTR-RT subfamilies (Table 2). The RPKM values showed variable copy numbers for 14 LTR-RT subfamilies in *Pyrus* accessions (Figure 2). *Gypsy-4_PX* had the highest RPKM value in every *Pyrus* species. In addition, *Copia-100_MAD*, *Copia-24_PX*, and *Copia-2_PX* have high copy numbers in *Pyrus* species. *P. betulaefolia* and *P. calleryana* had a low copy number of *Gypsy-3_PX*, but high copy numbers of *Copia-49_MAD*. For *Copia-106_MAD*, three cultivars (‘Suli’, ‘Qiushui’ and ‘Zaoshengxinshui’) had lower copy numbers than the wild pear accessions did (Figure 2).

**Figure 3:**
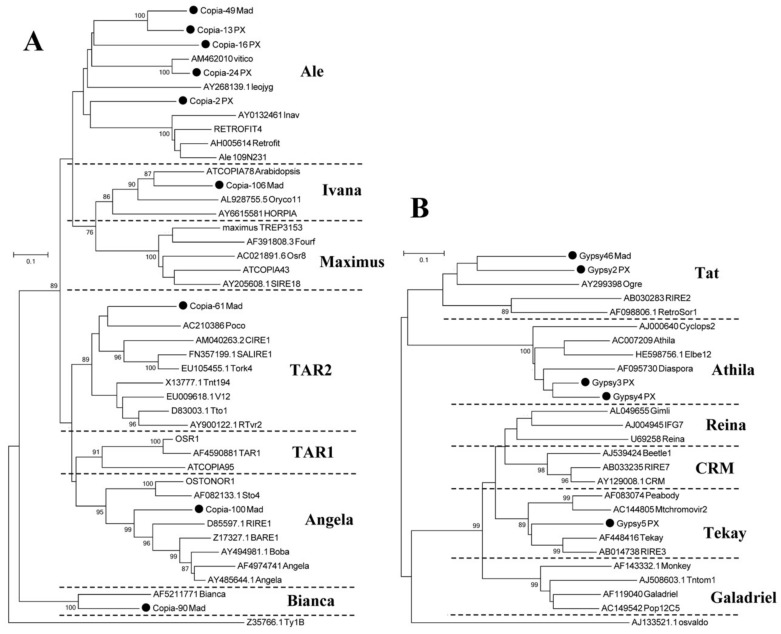
Phylogenetic relationships of reverse transcriptase sequences based on translated nucleotide sequences from 14 identified retrotransposons labeled with black dots. (**A**) Phylogenetic tree of *copia*-type reverse transcriptase sequences, including 17 identified reverse transcriptases. (**B**) Phylogenetic tree of *gypsy*-type reverse transcriptase sequences, including 22 identified reverse transcriptases.

The translated nucleotide sequences of the reverse transcriptases of 14 LTR-RT subfamilies were obtained, and these sequences were clustered with those of known TE families (Figure 3). The translated *copia*- and *gypsy*-type reverse transcriptase sequences clustered separately. Of the nine *copia*-type retrotransposons, five were clustered in the *Ale* family. The four remaining retrotransposons were clustered in the *TAR*, *Ivana*, *Angela*, and *Bianca* families, respectively (Figure 3). Among the *gypsy*-type retrotransposons, two were clustered in the *Tat* family, and two retrotransposons were clustered in the *Athila* family. The remaining one was clustered in the *Tekay* family (Figure 3).

### 3.5. The Insertion Times of the 14 LTR-RT Subfamilies in the ‘Suli’ Genome

**Figure 4:**
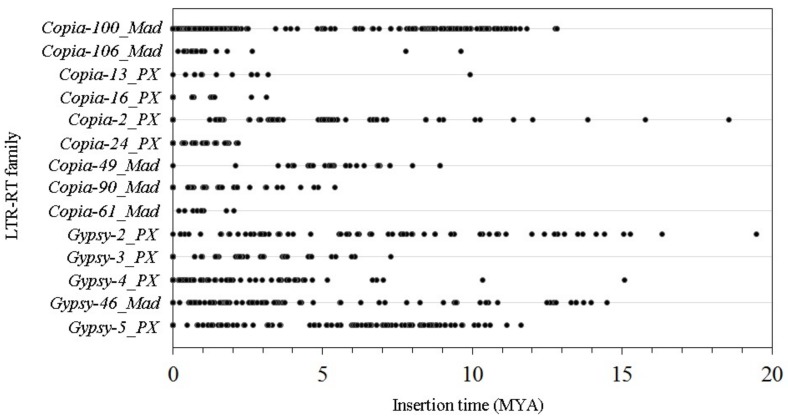
The insertion times of the 14 high-copy-number LTR-RT subfamilies in the ‘Suli’ genome.

The divergence of two LTRs was evaluated for the insertion times of LTR-RTs. We used a molecular clock rate of 4.72 × 10^−9^ substitutions per site per year [34]. For the 14 LTR-RT subfamilies, 1092 total pairs of LTRs were analyzed. The divergence of two nearly LTRs ranged from 0 to 0.18, representing a maximum insertion time of 19.49 million years ago (MYA). The predicted mean insertion times of the 14 LTR-RT subfamilies analyzed in this study was 3.42 MYA. Most LTR-RTs (92.5%) were estimated to have inserted into the genome during the last 10 million years (Figure 4). The peak copy number of retrotransposon mobilization (54.1%) was observed at 0–2 MYA, indicating that most of the isolated retrotransposons were relatively recently inserted.

### 3.6. The Insertion Sites of the 14 LTR-RT Subfamilies in the ‘Suli’ and ‘Bartlett’ Genomes

**Figure 5:**
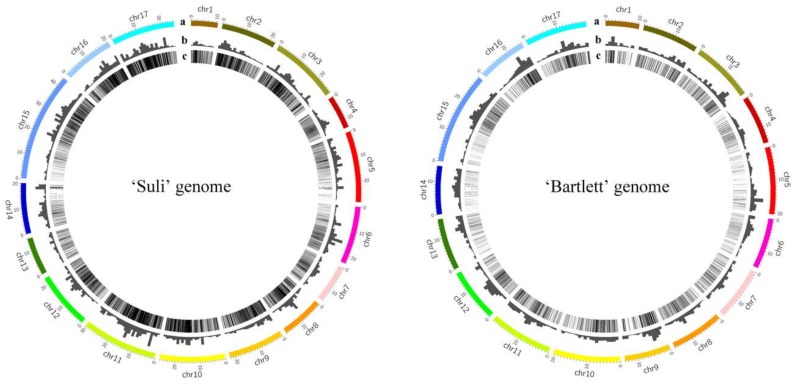
Distribution of the 14 LTR-RT subfamilies in the ‘Suli’ and ‘Bartlett’ genomes. (**a**) Chromosome. (**b**) Copy numbers of 14 LTR-RT subfamilies per Mb. (**c**) Distribution of 14 LTR-RT subfamilies.

Although the ‘Bartlett’ genome was poorly assembled with low coverage, information on its sequences could be obtained. To test the distribution of the 14 isolated LTR-RT subfamilies in *Pyrus* chromosomes, the positions of the retrotransposons in each chromosome were determined (Figure 5). A total of 28,901 and 6620 loci were tracked by BLASTN searching in the chromosomes of ‘Suli’ and ‘Bartlett’, respectively. In ‘Suli’, Chr11, Chr15 and Chr17 had more retrotransposons than other chromosomes (Supplementary Table S5). Chr1, Chr7 and Chr13 had fewer retrotransposons than others. In ‘Bartlett’, Chr15 had more retrotransposons than other chromosomes. In both ‘Suli’ and ‘Bartlett’ chromosomes, the high-copy-number LTR-RTs were not uniformly distributed (Figure 5). Aggregations of these LTR-RT subfamilies were found in some chromosome regions, such as 335 copies of LTR-RTs found in Chr11 (14-15 MB) in ‘Suli’.

**Figure 6:**
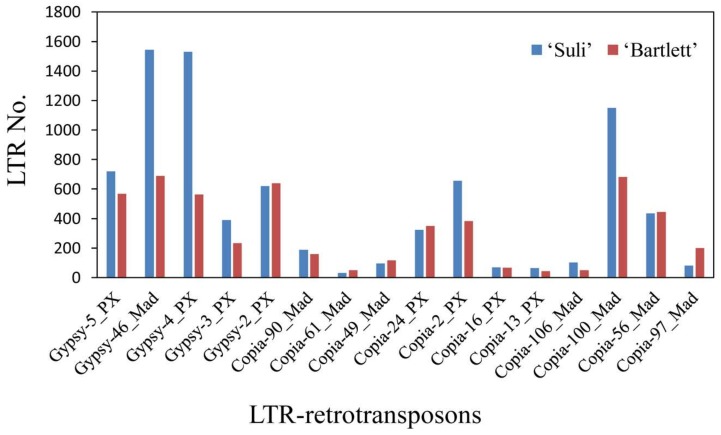
The number of LTR sequences of fourteen isolated LTR-RTs and two other selected LTR-RTs tracked in the ‘Suli’ and ‘Bartlett’ genomes.

The homologous and specific insertion sites of retrotransposons were evaluated in the evolution of *Pyrus*. The LTRs of isolated 14 LTR-RT subfamilies were blasted against the ‘Suli’ and ‘Bartlett’ genomes (Figure 6). The LTRs of *Copia-56_MAD* and *Copia-97_MAD* were also found many copies in the *Pyrus* genome, which was analyzed further. The results showed that almost all LTR-RT subfamilies had a higher number of LTRs in the ‘Suli’ genome than in the ‘Bartlett’ genome. Some LTRs were mapped to more than two hundred copies in the two pear genomes. *Gypsy-46_ MAD*, *Gypsy-4_ PX* and *Copia-100_MAD* had the highest number of LTRs in the ‘Suli’ genome. Most of the insertion sites of *Gypsy-5_PX* were the same in ‘Suli’ and ‘Bartlett’ genomes. Five LTRs were selected to search for homologous sites in two pear genomes (Table 3). Approximately 2504 and 1364 insertion sites were found in the ‘Suli’ and ‘Bartlett’ genomes, respectively. The insertion sites appearing in only one genome were considered specific sites. A total of 971 and 817 specific insertion sites were found in ‘Suli’ and ‘Bartlett’, respectively (Table 3).

## 4. Discussion

A large number of retrotransposons have been found in *Pyrus* and other plant genomes. More than ten thousand LTR-RT individuals were released in the Repbase database [30], which showed that the retrotransposons were heterogeneous. In this study, 4912 retrotransposons were identified by running LTR-harvest based on two nearly LTR flanking sequences in the ‘Suli’ genome. Some of them were mapped to partial sequences of reference retrotransposons (Supplementary Figure S1), which were not full-length retrotransposons. These findings indicated that the mutation of LTR-RTs in the ‘Suli’ genome was universal and variable, similar to those observed in rice, strawberry and Masson pine [36,37,38]. There could be several reasons for these mutations. First, gene mutations, including insertions and deletions, are major causes of heterogeneity. In recent reports, some retrotransposons were detected to exist before the speciation of *Pyrus* and *Malus* [39], and the insertion times of many retrotransposons date back more than 10 MYA [36]. Given the long genomic history of retrotransposons, potential sources of variation can relate to losses of the inner domains or mixture with other sequences, forming nonautonomous elements. These mutations would have accumulated over time, generating a highly heterogeneous population. Second, all transposons are integrated into chromosomal DNA. The mutation of active or nonfunctional retrotransposons is useless and is not regulated by natural selection. Therefore, mutated retrotransposon sequences, mainly nonsense mutations, could be transmitted to the next generation, thus maintaining a high degree of heterogeneity of retrotransposons between generations. Third, the sequencing technology of Illumina HiSeq 2000 restricted the assembly of the ‘Suli’ genome. Due to the short length of the reads (2 × 100 bp), repetitive sequences with long fragments were poorly assembled, which might cause the appearance of retrotransposon heterogeneity.

In previous research, the whole-genome sequencing of two pear genomes showed that a large percentage of the genome of the oriental pear ‘Suli’ and occidental pear ‘Bartlett’ were LTR-RTs [21,22], implying that retrotransposons may play important roles in *Pyrus* evolution. Because of the long genetic distance between oriental and occidental pear, the assembled genome of ‘Suli’ was chosen as the reference genome. Whole-genome resequencing showed that the percent of mapped reads in Asian pear accessions other than ‘Suli’ ranged from 41.9% to 50.9% (Table 1), suggesting that the genome sequences of Asian pears were slightly different. ‘Suli’ had a high percent of mapped reads (71.4%) because it was mapped to its own genome data. In Asian pear accessions, the total RPKM values of all isolated LTR-RT subfamilies revealed that the genomes of wild species had more LTR-RTs than those of cultivars did. The genome of *P. betulaefolia* had the largest number of LTR-RTs among Asian pears, suggesting that the insertion of LTR-RTs was active in the evolution of *P. betulaefolia*. Approximately 12.6% of LTR-RT subfamilies showed more than fourfold differences in the comparison between ‘Suli’ and *P. betulaefolia* (Figure 1B), suggesting that LTR-RTs differ greatly among Asian pears. A slight difference in the copy numbers of LTR-RTs was found between ‘Qiushui’ and ‘Zaoshengxinshui’. Both these samples belonged to *P. pyrifolia*. *P. betulaefolia* had a distant genetic relationship with the Asian cultivars, which was also reflected in the copy number changes of LTR-RTs.

The investigation of high-copy-number LTR-RT subfamilies was more representative. In the fourteen isolated high-copy-number LTR-RT subfamilies, six of them were annotated in *Malus*, suggesting that these subfamilies have existed in the *Pyrus* genome for a long time, since before the divergence of *Malus* and *Pyrus*. More than 50% of the LTR-RTs in the genomes of all *Pyrus* accessions were from these 14 identified LTR-RTs (Table 2), suggesting that these retrotransposons played critical roles in the evolution of *Pyrus*. *Gypsy-4_PX* had the highest copy number in each Asian pear, and this subfamily also exists in *Malus*, *Prunus* and *Fragaria*, implying that it might exist in other genera in the Rosaceae. The copy number of LTR-RT subfamilies differed among *Pyrus* species (Figure 2). Wild pear accessions had higher copy numbers of *Copia-106_MAD* than did the cultivars, implying that this subfamily has been duplicated many times in wild accessions or lost a lot in cultivars. In addition, *Gypsy-46_MAD* and *Gypsy-5_PX* had higher copy numbers in ‘Suli’ and *P. pashia* than in other accessions. These changes in the copy numbers of retrotransposon subfamilies might cause genetic divergence in *Pyrus* species. *Copia-2_PX*, *Gypsy-4_PX*, *Copia-24_PX* and *Copia-100_MAD* had high copy numbers in the genomes of all accessions, suggesting that these LTR-RTs might be still active and account for a large proportion of the *Pyrus* genome. The classification of LTR-RTs showed that the high-copy-number *copia*-type retrotransposons derived mainly from the *Ale* family, suggesting that this family was important in the evolution of the *copia* retrotransposon in the *Pyrus* genome. Four LTR-RTs, *Gypsy-46_MAD*, *Gypsy-2_PX*, *Gypsy-3_PX* and *Gypsy-4_PX*, were from the *Tat* and *Athila* family, in accordance with a result showing that many *gypsy*-type retrotransposons in the ‘Suli’ genome were derived from these two families in a previous study [39].

The average insertion time of the 14 high-copy-number LTR-RT subfamilies in the ‘Suli’ genome was 3.42 MYA, suggesting that many LTR-RTs were recently inserted into the genome. Approximately 54.1% of LTR-RTs from high-copy-number subfamilies were transposed at 0-2 MYA (Figure 4), implying a recent peak in mobilization. Several LTR-RTs were still active and continuously being inserted into the *Pyrus* genome. Retrotransposons are involved in changing the sizes of genomes by either increasing the genome size or promoting rapid genomic DNA loss [5,6]. In *Pyrus*, genome size did not greatly vary among species [40,41]. In the ‘Suli’ genome, a large number of LTR-RTs were matched to partial sequences of reference retrotransposons, suggesting that these LTR-RTs had lost some fragments, promoting genomic DNA loss. However, the insertion times showed that most LTR-RTs had been duplicated many times in recent years, thereby increasing the genome size. We inferred that a balance had existed between genome expansion and contraction during the evolution of *Pyrus* for a long time. The distribution of LTR-RTs in the chromosomes could reveal the structure of the *Pyrus* genome. In this study, the 14 isolated LTR-RT subfamilies were nonuniformly distributed in the ‘Suli’ and ‘Bartlett’ genomes (Figure 5). Some regions of the chromosome had a large number of LTR-RTs, suggesting that the LTR-RTs might insert easily in these regions.

In this study, many specific insertion sites were found in oriental and occidental pears, implying that the insertion of LTR-RTs occurred during the evolution of *Pyrus*. Furthermore, many homologous insertion sites were also found, suggesting that these insertion sites had existed in oriental and occidental pears for a long time. In *Gypsy-5_PX*, most of the insertion sites were the same in the ‘Suli’ and ‘Bartlett’ genomes, implying that this subfamily had duplicated many times before the divergence of oriental and occidental pears. In *Gypsy-4_PX*, many specific insertion sites were found in the ‘Suli’ and ‘Bartlett’ genomes, suggesting that *Gypsy-4_PX* had duplicated many times after the divergence of oriental and occidental pears. The presence of both homologous and specific insertion sites suggested that the duplication of LTR-RTs had occurred almost constantly throughout the origin and evolution of *Pyrus* species.

## 5. Conclusions

A large number of LTR-RTs was identified from the *Pyrus* genome, and fourteen high-copy-number LTR-RTs were isolated in six Asian pear accessions. The resequencing data showed that these 14 identified LTR-RTs in the Asian pear genomes represented more than 50% of the retrotransposon dataset. Some of these sequences have existed in *Pyrus* species for a long time and rapidly expanded during the last 3.42 million years, after the divergence of *Malus* and *Pyrus*. Among the six Asian pear accessions, *P. betulaefolia* had the highest number of LTR-RTs in its genome. Of all the subfamilies, *Gypsy-4_PX* accounted for the highest proportion in the genomes of Asian pears. To the best of our knowledge, this study is the first to use the technology of resequencing to identify high-copy-number LTR-RTs in plants. The high copy number and diversity of LTR-RT subfamilies in Asian pears demonstrate the importance of retrotransposons as a source of genetic variation in *Pyrus* genomes.

## Figures and Tables

**Table 1:** The number of processed reads and mapped reads in this study.

	‘Suli’ *	‘Qiushui’	‘Zaoshengxinshui’	*P. pashia*	*P. calleryana*	*P. betulaefolia*
Reads processed (M)	296.7	45.5	50.1	57.4	49.5	51.8
Mapped reference genome reads (M)	207.7 (71.4%)	23.2 (50.9%)	24.4 (48.9%)	24.1 (41.9%)	22.4 (45.3%)	22.5 (43.5%)
Mapped 4,912 LTR-RTs reads (M)	49.5	5.2	5.5	6.7	6.7	7.9
Mapped LTR-RTs/mapped reference genome	23.8%	22.4%	22.5%	27.8%	29.9%	35.1%

* The clean reads of ‘Suli’ were downloaded from the NCBI database.

**Table 2:** The RPKM values of the 14 long terminal repeat retrotransposons (LTR-RT) subfamilies and all isolated LTR-RTs.

	‘Suli’	‘Qiushui’	‘Zaoshengxinshui’	P. pashia	P. calleryana	P. betulaefolia
RPKM value of 14 LTR-RT subfamilies	396.5	337.1	326.2	508.8	545.2	625.0
RPKM value of 198 LTR-RT subfamilies	700.6	589.8	596.2	800.0	790.0	863.7
Percent (%)	56.6	57.1	54.7	63.6	69.0	72.4

**Table 3:** The specific insertion sites in ‘Suli’ and ‘Bartlett’ based on five isolated LTR-RTs.

	Mapped Sequences in ‘Suli’	Specific Insertion Sites in ‘Suli’	Mapped Sequences in ‘Bartlett’	Specific Insertion Sites in ‘Bartlett’
*Gypsy-2_PX*	412	241	396	232
*Gypsy-3_PX*	234	97	86	58
*Gypsy-4_PX*	1254	404	468	330
*Copia-24_PX*	302	145	247	160
*Copia-56_MAD*	302	84	167	37
Total	2504	971	1364	817

## Supplementary Materials

The following are available online at http://www.mdpi.com/2073-4425/10/2/156/s1, Figure S1: The complex composition of the LTR-RTs AJSU01004483.1_13452_15649 and AJSU01000586.1_13421_21925 in the ‘Suli’ genome, Table S1: The classification and value of the RPKM in all 1,276 LTR- retrotransposons, Table S2: The value of RPKM in all 198 LTR-RT families, Table S3: The value of RPKM of the 198 LTR-RT families in three sequencing data (SRR609906, SRR609907 and SRR609912) of ‘Suli’, Table S4: The high copy number of LTR-retrotransposon families, Table S5: The number of 14 identified LTR-RTs in 17 chromosomes in ‘Suli’ and ‘Bartlett’.

## Abbreviations

LTR-RT	long terminal repeat retrotransposon
RPKM	reads per kilobase per hundred thousand mapped pear reference genome (‘Suli’ genome) reads
